# Singular Pompeiian Statues

**Published:** 1871-07

**Authors:** 


### THE DOCTOR.

He must be a man of the strictest honor and integrity, for to him are confided the secrets of families, the honor of wives and daughters—the secret trusts that are committed to no other. Every medical man should feel the responsibility of these trusts, or he is unworthy of being a physician. The physician who practices his profession merely as a trade for the amount of money that can be made by it, is unworthy of his calling. The higher and nobler motive of doing good to others, of relieving human suffering, of prolonging human life, is the only incentive that ever has or ever will make the great physician. In proportion to the weight of his responsibility should be the honor and the integrity of his character. How easy it is for the physician to control the destiny of his patients. On him they rest, and confide in his knowledge and truth. He decides for them questions of life and death. Happiness or unhappiness it is in his power to give, and why? The greater his knowlędge the greater is his power. He has knowledge of how to do good, and consequently the power to do evil; and therefore the necessity of his being governed by the strictest honor and integrity in order to use that knowledge rightly.—*Prof. L. A. Sayre, New York.*

HOW MUCH TO EAT.—One of the questions oftenest asked me is: "How much shall I eat?" It is a question which each one must answer for himself. The best general guide in this matter, is to avoid eating enough to make you feel dull and sleepy, or to produce any unpleasant sensations in the head or stomach. If such feelings are experienced after meals, you may know you have eaten too much, and that you must eat less thereafter. If, as is the case with many persons, you can not control the amount in any other way, and stop when you have eaten enough, you should measure out the amount you are going to eat, and put it upon your plate before you begin, and never allow yourself to be tempted to take any thing more. There is very little danger of not eating enough. There are such cases, however, but they are confined to the class of persons who lead a sedentary, in-door life. If such persons will change their mode of life, and take as much out-door exercise as they are able to, their appetite will return, and they will soon eat a sufficient quantity.—*Herald of Health.*

### Singular Pompeiian Statues.

During the eruption of Vesuvius, which destroyed the ancient city of Pompeii, those who delayed too long in making their escape, fell victims, for the most part, to the pernicious effects of sulphuric and carbonic acid gases, and were rapidly covered with the showers of fine dust following the eruption, which, gradually hardening, formed perfect moulds of the unhappy beings who so miserably perished, from which admirable casts are taken, showing their forms, features, expression, and attitude when overtaken by death. At the beginning of the excavations, little attention was paid to the natural moulds, only a few having been partially cast and preserved, the most remarkable of which were those of a husband, wife and child; the husband at the moment of death pressing tightly to his breast nineteen pieces of gold and ninety-one pieces of silver, which were found fixed to his ribs; the wife had let fall a coarse linen cover in which were found fourteen bracelets, gold ear-rings, and jewels of less importance. It was only, however, in 1863, that M. Fiorelli had the happy idea of filling those natural moulds with a peculiar solution of plaster by which process the victims are reproduced in their integrity.

The first group reproduced was composed of a man, a woman, and two young girls, who had remained within doors until too late; when they attempted to escape by the window or terrace, they were suddenly asphyxiated, and covered with the dust, which faithfully preserved the contour of their forms. In 1868, a body thus reproduced was that of a man who had fallen face downward, whose countenance was the very image of despair and suffering, his clinched teeth and clasped hands eloquently expressing the agony he had endured. Next in interest is the form of a woman who had fallen on her back, whose right hand leans upon the earth, her left hand raised, as if trying to ward off the danger. To aid her flight she had raised her vestments. Her form is tall and elegant, her admirably arched foot incased in strong sandals, being a favorite subject of study to artists. On one of her fingers is a silver ring, while near her were found gold ear-rings, a silver mirror, and an amber statue, representing Cupid. Her hair in the front forms three rows of ringlets, and falls plaited over her back in the manner of the Voltarie *perruques*.

A remarkable group of three persons has been admirably cast, which is in the highest degree interesting. A man of tall statue and powerful build, and strongly marked features, prominent cheek bones, heavy beard and mustache, is the principal figure; he held in his hands the ear-rings of the two young girls who had followed him, and the key of the house, and the *beau ideal* of an old Roman legionary. Over his head he had thrown the corner of his mantle for protection against the noxious gases or the falling dust and cinders, the expression on his face and that of his two daughters being suggestive of suffocation. There is something touching in the spectacle of the two sisters who followed their father in the precise attitude as they fell, supporting each other, breathing the same poison, and dying entwined in each other's arms. Both the figures are of beautiful forms and proportions.—*Appleton's Journal.*

HIS OWN GRANDFATHER.—We clip the following amusing article from an exchange. It illustrates the evils of intermarriage, showing that a fellow can get so thoroughly mixed up as to be unable even to recognize himself. This is the condition of the young man who tells the following harrassing story:—

"I married a widow who had a grown up daughter. My father visited my house very often, fell in love with my step-daughter, and married her. So my father became my own son-in-law, and my step-daughter became my step-mother, because she was my father's wife. Some time afterward my wife had a son; he was brother of my step-mother. My father's wife, that is, my step-daughter also had a son; he was, of course, my brother, and in the meantime my grandchild, for he was the son of my daughter. My wife was my grandmother, because she was my step-mother's mother. I was my wife's husband and grandchild at the same time. And as the husband of a person's grandmother is the grandfather, I was my own grandfather."

A New York paper tells of a cat, that every morning wipes his paws upon the hall mat before entering his mistress's bedroom. If his feet leave a mark on the white coverlet of the bed, he is told of the fact, and again resorts to the mat, and then if not satisfied that his paws are clean, he dries them by the stove.

A physician writes asking a renewal of a note which he owes, giving as a reason therefor: "We are in a horrible crisis; there is not a sick man in the district."

